# The Effect of 14-Day Consumption of Hydrogen-Rich Water Alleviates Fatigue but Does Not Ameliorate Dyspnea in Long-COVID Patients: A Pilot, Single-Blind, and Randomized, Controlled Trial

**DOI:** 10.3390/nu16101529

**Published:** 2024-05-19

**Authors:** Yineng Tan, Yixun Xie, Gengxin Dong, Mingyue Yin, Zhangyuting Shang, Kaixiang Zhou, Dapeng Bao, Junhong Zhou

**Affiliations:** 1 School of Strength and Conditioning Training, Beijing Sport University, Beijing 100084, China; 2023210116@bsu.edu.cn; 2 College of Swimming, Beijing Sport University, Beijing 100084, China; yixunxie00@163.com; 3 School of Sport Medicine and Physical Therapy, Beijing Sport University, Beijing 100084, China; ddgx0419@163.com; 4 School of Athletic Performance, Shanghai University of Sport, Shanghai 200438, China; mingyue0531@sus.edu.cn; 5 College of Physical Education and Health Management, Chongqing University of Education, Chongqing 400065, China; shangzhang0301@163.com; 6 College of Physical Education and Health Science, Chongqing Normal University, Chongqing 401331, China; 7 China Institute of Sport and Health Science, Beijing Sport University, Beijing 100084, China; 8 Hebrew Senior Life Hinda and Arthur Marcus Institute for Aging Research, Harvard Medical School, Boston, MA 02115, USA; junhongzhou@hsl.harvard.edu

**Keywords:** Long-COVID, hydrogen-rich water, fatigue, dyspnea

## Abstract

(1) Background: Hydrogen (H_2_) may be a potential therapeutic agent for managing Long COVID symptoms due to its antioxidant and anti-inflammatory properties. However, more scientific literature is needed to describe the effects of H_2_ administration on treating symptoms. A study aimed to investigate the impact of hydrogen-rich water (HRW) administration on the fatigue and dyspnea of Long-COVID patients for 14 consecutive days. (2) Methods: In this randomized, single-blind, placebo-controlled study, 55 participants were recruited, and 23 of them were excluded. A total of 32 eligible participants were randomized into a hydrogen-rich water (HRW) group (*n* = 16) and a placebo water (PW) group (*n* = 16) in which they were instructed to consume hydrogen-rich water or placebo water for 14 days, respectively. The participants completed the Fatigue Severity Scale (FSS), Six-Minute Walk Test (6MWT), 30 s Chair Stand Test (30s-CST), Modified Medical Research Council Dyspnea Rating Scale (mMRC), Pittsburgh Sleep Quality Index (PSQI), and depression anxiety stress scale (DASS-21) before and after the intervention. A linear mixed-effects model was used to analyze the effects of HRW. Cohen’s d values were used to assess the effect size when significance was observed. The mean change with 95% confidence intervals (95% CI) was also reported. (3) Results: The effects of HRW on lowering FSS scores (*p* = 0.046, [95% CI = −20.607, −0.198, d = 0.696] and improving total distance in the 6WMT (*p* < 0.001, [95% CI = 41.972, 61.891], d = 1.010), total time for the 30s-CST (*p* = 0.002, [95% CI = 1.570, 6.314], d = 1.190), and PSQI scores (*p* = 0.012, [95% CI = −5.169, 0.742], d = 1.274) compared to PW were of a significantly moderate effect size, while there was no significant difference in mMRC score (*p* = 0.556) or DASS-21 score (*p* > 0.143). (4) Conclusions: This study demonstrates that HRW might be an effective strategy for alleviating fatigue and improving cardiorespiratory endurance, musculoskeletal function, and sleep quality. Still, it does not ameliorate dyspnea among Long-COVID patients.

## 1. Introduction

Long COVID, or “Post-COVID condition”, is typically characterized by several severe symptoms (e.g., fatigue and dyspnea) following SARS-CoV-2 infection [1,2]. It is important to note that these symptoms may persist even after a negative COVID-19 test. Therefore, developing appropriate strategies for helping Long-COVID patients manage (e.g., fatigue and dyspnea) and recover from these symptoms is crucial.

Long-COVID symptoms are linked to immune dysregulation due to viral persistence, which may cause impaired physical health and chronic inflammation [3]. Cytokine signaling plays a crucial role in this disease’s pathogenesis by triggering a chain reaction of oxidative stress and inflammation [4,5]. These dysregulations and exceedingly high oxidative stress and inflammation levels may thus lead to persistent fatigue and dyspnea [6,7]. Additionally, these symptoms may be accompanied by a deterioration in physical ability (e.g., cardiorespiratory endurance and musculoskeletal function) [8]. Therefore, strategies aimed at reducing oxidative stress and inflammasome activation may help alleviate the fatigue and dyspnea induced by Long COVID and symptoms related to these conditions (i.e., decreased cardiorespiratory endurance and musculoskeletal function) [9,10].

Increasing hydrogen (H_2_) intake is one such strategy, offering significant antioxidative and anti-inflammatory properties, which may provide an advantage in fatigue alleviation [11,12]. Recent studies have suggested that taking in H_2_ in the form of gas and water (i.e., hydrogen-rich water, HRW) can help alleviate the symptoms (e.g., dyspnea) of COVID-19 [13,14]. For example, a study demonstrated that HRW may improve oxygen saturation and exercise tolerance in COVID-19 patients, potentially reducing hypoxic episodes when engaging in physical activity [15]. Additionally, a recent study showed, in a group of forty-four COVID-19 patients, that inhaling hydrogen-rich gas until discharge could significantly alleviate the severity of respiratory symptoms and minimize inspiratory resistance [16]. Still, the effects of taking in H_2_ on fatigue and dyspnea in the Long-COVID phase, and the related cardiorespiratory endurance and musculoskeletal function, have not been examined.

Therefore, in this pilot, randomized, single-blind study, we examined the effects of consuming HRW twice daily for consecutive 14 days on fatigue, dyspnea, cardiorespiratory endurance, and musculoskeletal function in individuals with Long-COVID. To the best of our knowledge, this is the first study demonstrating the effects of HRW intake on symptoms in people suffering from Long COVID. We hypothesized that, compared to the controls (who consumed placebo water), the participants who consumed HRW would have greater reductions in the severity of fatigue and dyspnea, and such alleviation would be associated with greater improvement in cardiorespiratory endurance and musculoskeletal function.

## 2. Methods

### 2.1. Participants

**Figure 1:**
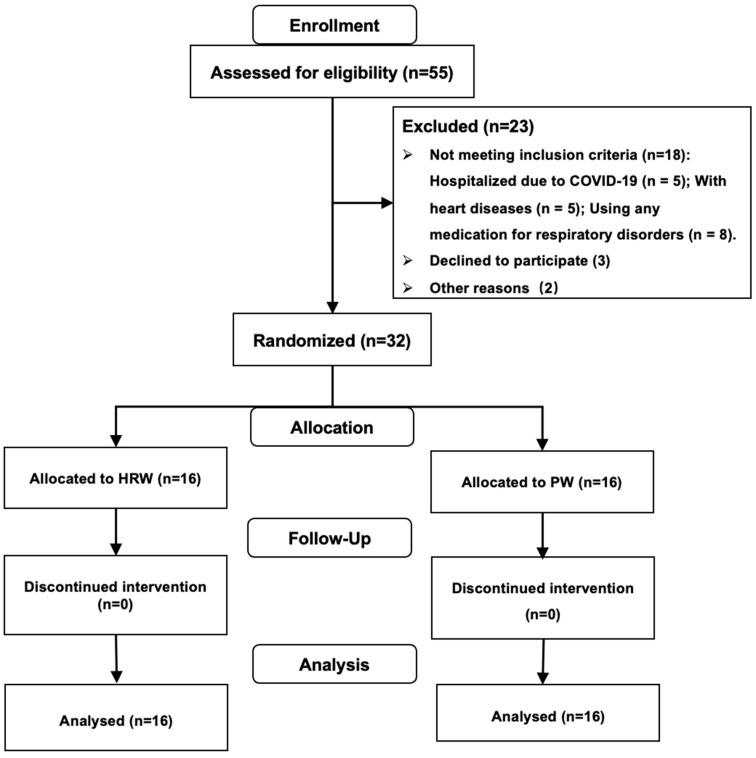
The Consolidated Standards of Reporting Trials (CONSORT) diagram. HRW, hydrogen-rich water group; PW, placebo water group.

Participants were recruited remotely through an online questionnaire applet and e-posters. The inclusion criteria were (1) being between 18 and 65 years of age; (2) having been diagnosed with SARS-CoV-2 infection 21–35 days prior to participation and self-reported heightened fatigue and dyspnea compared to the pre-infection period for longer than four weeks after the diagnosis of COVID-19 [17]; (3) having received at least two doses of the COVID-19 vaccine before infection with COVID-19; (4) a resting oxygen saturation level of ≥95%, verified using a smartwatch (HUAWEI Watch 3, Goertek, Weifang, China) [18]; and (5) willingness to complete all the study procedures. The exclusion criteria included (1) hospitalization due to COVID-19 and/or (2) affliction with significant endocrine disorders, acute diseases, heart diseases, uncontrolled high blood pressure, or other neurological disorders; current use of any medication for respiratory disorders; and pregnancy or lactation (Figure 1). The Research Ethics Committee of Beijing Sport University approved the study protocol (Approval number: 2023301H), and all procedures were conducted according to the Declaration of Helsinki. Before the experiment, participants were informed of the benefits and potential risks related to the study, and all signed an informed consent form.

### 2.2. Study Protocol

**Figure 2:**
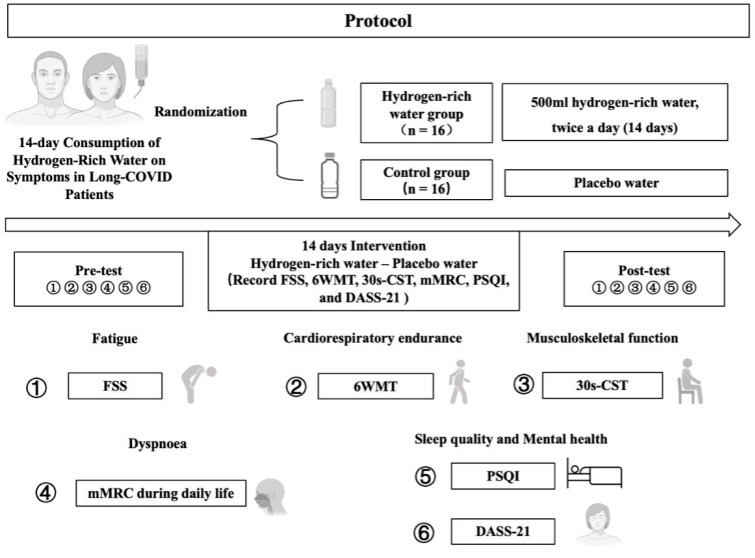
Overview of protocol. FSS, fatigue severity scale; 6MWT, six-minute walk test; 30s-CST, 30 s chair stand test; mMRC, modified medical research council dyspnea scale; PSQI, Pittsburgh sleep quality index; DASS-21, depression anxiety stress scale.

At baseline and immediately (on the same day) after the last session of intervention, we measured fatigue, dyspnea, cardiorespiratory endurance, and musculoskeletal function in the patients’ homes. Familiarization was completed before the study, and all the home-based assessments were administrated by the study staff remotely. Considering that sleep quality and mood, which may also be significantly altered after COVID-19, contributed to fatigue and dyspnea (Figure 2), we measured them in this work.

### 2.3. Intervention Protocol

Participants were randomly assigned to either the HRW group (*n* = 16) or the PW group (*n* = 16) using a random number generator (version 2021, Microsoft Excel) for 14 days of intervention (Figure 2), utilizing the ‘RAND ()’ function to generate a random number between 0 and 1 for each participant. The sample size was determined using G-Power (version 3.1.9.7; Franz Faul, University of Kiel, Kiel, Germany) by using an err prob = 0.05, 1 − βErr Prob = 0.8, and an effect size of f = 0.26; and the test family = F test. The personnel who administrated the assessments were aware of each participant’s group assignment. The personnel who recorded clinical and laboratory data were blinded to the groups. The HRW group drank 500 mL of HRW daily in the morning and evening, with the HRW being consumed immediately after preparation (i.e., within 10 min) to ensure the hydrogen level was as designed and had not decreased. The PW group consumed an equal amount of pure water. The HRW used (H_2_ concentration: 1600 ppb; pH: 7.6; ORP: −590 mv; Temperature: 22 °C) was prepared using an electrolysis device (Zhiheng Hydrogen Health Technology Co., Ltd., Fuzhou, China) with a transparent Tritan^TM^ tank body and an electrolysis generator. To ensure blinding, both groups received water contained in identical packaging.

Participants maintained their usual lifestyle and diets, refraining from consuming alcohol and engaging in strenuous activity 48 h before physical tests. They also avoided consuming caffeine and substances that could affect test results on the testing day. Tests were conducted between 8:00 a.m. and 11:00 a.m. to minimize circadian rhythm effects, and participants abstained from consuming HRW on the testing day to ensure result accuracy. None of the participants smoked or took any supplements/drugs during the experiment.

#### 2.3.1. Assessment of Fatigue

Fatigue Severity Scale (FSS). We used FSS to measure the severity of fatigue in Long-COVID patients. Participants rated their agreement on a scale of 1 to 7 (1 = “strongly disagree”; 7 = “strongly agree”) [19]. Lower values indicate strong disagreement, while higher scores represent greater fatigue.

#### 2.3.2. Assessment of Dyspnea

The Modified Medical Research Council Dyspnea Scale (mMRC). We used a five-level description of mMRC to measure the severity of dyspnea in daily life. Level 0 denotes no dyspnea, while level 4 represents severe dyspnea, even at rest [20].

#### 2.3.3. Assessment of Cardiorespiratory Endurance

The 6-Minute Walk Test (6MWT). The 6MWT was used to evaluate cardiorespiratory endurance. It has been widely employed for evaluating chronic respiratory conditions and COVID-19 patient classification [21]. This test involves walking back and forth between markers placed 30 m apart in a wide, flat area for six minutes. The total distance participants walked (i.e., the total distance specified for the 6WMT) was recorded and used in the following analysis.

#### 2.3.4. Assessment of Musculoskeletal Function

The 30-second Chair Stand Test (30s-CST). We used 30s-CST to assess the musculoskeletal function of Long-COVID patients [22]. To perform this test, each participant was asked to stand up from a chair with their arms crossed on their chests and then return to a sitting position as many times and as safely as possible within 30 s. The number of times each participant was able to complete the sit-to-stand action was used in the following analysis [23].

#### 2.3.5. Assessment of Sleep Quality and Mood

The Pittsburgh Sleep Quality Index (PSQI). We used PSQI to measure the sleep quality of Long-COVID patients over one month. In this test, seven dimensions are scored, yielding an overall sleep quality/disturbance rating. Higher scores indicate poorer sleep quality; a global sum of 5 or greater indicates a “poor” sleeper [24].

The Depression, Anxiety, and Stress Scale (DASS-21). This scale comprises 21 items, encompassing three subscales for depression, anxiety, and stress, each containing seven items for measuring mood in a post-COVID-19 World [25]. It was designed to assess Long-COVID patients’ psychological states over a specific period, typically the past week. A greater score reflects more serious mood issues.

### 2.4. Study Outcomes 

FSS score, mMRC score, total distance in 6WMT, and total times in 30s-CST were the primary outcomes for fatigue, dyspnea, cardiorespiratory endurance, and musculoskeletal function, respectively. We also calculated the percent changes of these outcomes from pre- to post-intervention for our secondary analyses, as described in the Section 2.5.

### 2.5. Statistical Analysis

The experimental data were processed using the IBM SPSS statistical package (version 25.0, IBM Statistics, Chicago, IL, USA). Data were presented as means ± standard deviations (M ± SD). The mean change with 95% confidence intervals (95% CI) was also reported. At baseline, the differences in demographics (i.e., age, weight, height, and BMI) and outcomes (i.e., FSS, mMRC, 6MWT, 30s-CST, PSQI, and DASS-21) between groups were examined using linear mixed-effects model. 

We used linear mixed-effects models to examine the effects of the intervention on each primary outcome (i.e., FSS score, mMRC score, total distance in 6WMT, and total times in 30s-CST). The dependent variable for these models was each of these primary outcomes. The model factors were group (HRW and PW), time (pre- and post-intervention), and their interaction. Age, gender, BMI, sleep quality, anxiety, and stress were included as covariates. 

Secondarily, linear mixed-effect models were used to examine the difference in the percent change of each outcome (i.e., FSS score, mMRC score, total distance of 6MWT, total times of 30s-CST) between groups. The model factor was groups (i.e., HRW and PW). Age, gender, BMI, sleep quality, and anxiety and stress were included as covariates in these models.

Considering the variance in sleep quality, anxiety, and stress among the included participants (see Table 1), we also performed exploratory subgroup analyses on those who had difficulty sleeping or anxiety and depression. As described above, the models used for all participants were used for these exploratory subgroup analyses.

To examine the relationship between the changes in fatigue and dyspnea as induced by HRW and those in cardiorespiratory endurance and musculoskeletal function, we used linear regression models. We focused on the outcomes with significant changes induced by HRW for the HRW group.

All individuals were included in the intention-to-treat analyses, and missing data were not imputed. For all statistical tests, *p* < 0.05 was considered statistically significant. In addition to statistical significance, Cohen’s d (d) (classified as follows: 0.20~0.60 indicates small, 0.60~1.20 indicates moderate, 1.20~2.0 indicates large, and ≥2.0 indicates extremely large) were used to assess the size of the effect for which significance was observed [26].

## 3. Results

A total of fifty-five participants were initially remotely recruited. Thirty-two of them were eligible and then randomly assigned to either the hydrogen-rich water (HRW) group (33.38 ± 11.05 years, 170.06 ± 11.71 cm, 67.60 ± 15.19 kg, 23.16 ± 2.95 kg/m^2^, *n* = 16) or the placebo water (PW) group (38.31 ± 14.52 years, 167.25 ± 8.57 cm, 64.91 ± 9.30 kg, 23.20 ± 2.82 kg/m^2^, *n* = 16) (Figure 1). All participants completed this study, and their data were included in the analysis. No significant differences between groups were observed in terms of age, body weight, height, and BMI (Table 1). Additionally, nineteen participants experienced difficulty sleeping (HRW = 9, PW = 10), and sixteen participants had anxiety and depression (HRW = 9, PW = 7).

### 3.1. Effects of HRW on Fatigue

The primary linear mixed-effects model showed significant main effects of time (*p* = 0.001) but not group (*p* = 0.122) and interaction (*p* = 0.184) on FSS score (Table 2). Secondarily, the linear mixed-effects model showed that the percent change in FSS score (*p* = 0.046, [95% CI = −20.607, −0.198], d = 0.696) from baseline to post-intervention was significantly greater in the HRW group compared to the PW group. No significant effects of the covariates (*p* = 0.245~0.939) were observed.

### 3.2. Effects of HRW on Dyspnea

The linear mixed-effects model showed significant main effects of time (*p* = 0.032), but not group (*p* = 0.552) and interaction (*p* = 0.323), on mMRC score.

Secondarily, the linear mixed-effects model showed that the percent change in mMRC score from baseline to post-intervention was not significantly different in the HRW group compared to the PW group (*p* = 0.556).

### 3.3. Effects of HRW on Cardiorespiratory Endurance

The linear mixed-effects model showed no significant interactions between group and time (*p* = 0.279), as well as the main effects of time (*p* = 0.061) and group (*p* = 0.279), on the total distance in the 6WMT.

Secondarily, the linear mixed-effects model showed that the percent change in the total distance in the 6WMT (*p* < 0.001, [95% CI = 41.972, 61.891], d = 1.010) from baseline to post-intervention was significantly greater in the HRW group compared to the PW group. No significant effects of the covariates (*p* = 0.105~0.998) were observed.

### 3.4. Effects of HRW on Musculoskeletal Function

The linear mixed-effects model showed significant main effects of time (*p* = 0.015), but not group (*p* = 0.273) or interaction, on total times in the 30s-CST (*p* = 0.221).

Secondarily, the linear mixed-effects model showed that the percent changes in the total times in the 30s-CST (*p* = 0.002, [95% CI = 1.570, 6.314], d = 1.190) from baseline to post-intervention were significantly different in the HRW group compared to the PW group. No significant effects of the covariates (*p* = 0.248~0.876) were observed.

### 3.5. The Association between Fatigue, Cardiorespiratory Endurance, and Musculoskeletal Function

Based upon the results described above, we developed linear regression models for the associations between the percent changes in fatigue (i.e., the change in FSS score) and cardiorespiratory endurance (i.e., the change of total distance of 6WMT) and musculoskeletal function (i.e., the change of total times of 30s-CST) within the HRW group.

**Figure 3:**
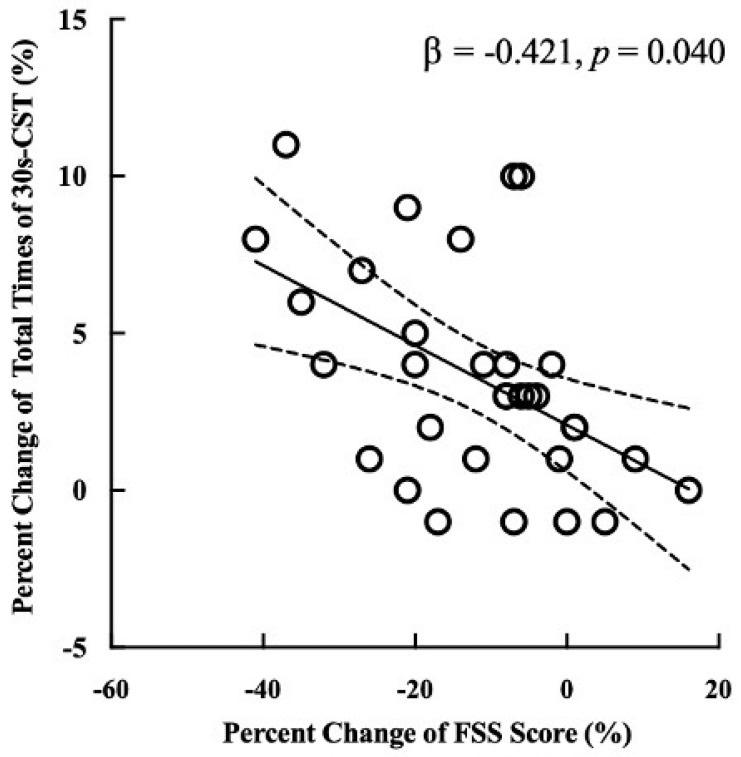
The association between the percent changes in fatigue (i.e., the percent change in FSS score) and musculoskeletal function (i.e., the percent change in total times in the 30s-CST) within the HRW group. The solid line represents the regression line showing the trend in data. Dashed lines indicate the 95% confidence intervals. The percent change in FSS score was significantly associated with the percent change in total times for the 30s-CST (β = −0.421, *p* = 0.040). Participants with greater fatigue alleviation (i.e., greater percent reductions in FSS score) exhibited greater improvement in musculoskeletal function (i.e., greater percent increases in total times for the 30s-CST). FSS, fatigue severity scale; 30s-CST, 30 s chair stand test.

It was observed that within the HRW group, the percent change in FSS score was significantly associated with the percent change in total time for the 30s-CST (β = −0.421, *p* = 0.040) (Figure 3). Specifically, participants with greater fatigue alleviation (i.e., changes in FSS score) had greater improvements in musculoskeletal function (i.e., changes in total time for the 30s-CST). No significant association between the percent change in FSS score and the percent change in total distance for the 6WMT (*p =* 0.975) was observed.

### 3.6. Effects of HRW on Sleep Quality and Mood: Exploratory Subgroup Analysis

For those with difficulty sleeping, the exploratory subgroup analyses showed significant main effects of time (*p* = 0.011) and marginally significant effects of interaction (*p* = 0.064), but not group (*p* = 0.909), on sleep quality as assessed via PSQI score (Table 3).

Secondarily, it was observed that the percent change in PSQI score (*p* = 0.012, [95% CI = −5.169, 0.742], d = 1.274) from baseline to post-intervention was significantly greater in the HRW group compared to the PW group. No significant effects of the covariates (*p* = 0.407~0.558) were observed.

For those with stress and anxiety, no significant effects of time (*p* = 0.121~0.314), group (*p* = 0.443~0.909), or interaction (*p* = 0.602~0.723) on depression score, anxiety score, or stress score were observed. Similarly, no significant differences in the percent changes for these outcomes (*p* = 0.441~0.657) from baseline to post-intervention were observed between the HRW and PW groups.

## 4. Discussion

The findings of this pilot study demonstrate that the daily intake of HRW provides promising benefits for alleviating fatigue and helps improve cardiorespiratory endurance, musculoskeletal function, and sleep quality among people suffering from Long COVID.

It was observed that HRW induced significantly greater fatigue alleviation than the control, and greater fatigue alleviation is associated with greater improvement in musculoskeletal function. This is consistent with previous studies showing that H_2_ can alleviate fatigue among healthy individuals and those with chronic fatigue syndrome [27,28,29,30]. Several potential mechanisms may contribute to such benefits. First, studies showed that H_2_ can reduce inflammation and neutralize excess oxygen-free radicals, helping alleviate fatigue [31,32]. Second, H_2_ may suppress the generation of superoxide in mitochondrial complex I by altering electron transport within the inner membrane of a mitochondrion, alleviating oxidative stress and ultimately leading to decreased fatigue caused by excessive levels of oxidative and nitrosative stress [33,34]. This may then promote mitochondrial multiplication and help increase muscle energy efficiency, which ultimately helps improve musculoskeletal function. Still, direct evidence of the underlying neurophysiological pathways through which HRW intake can help fatigue alleviation in Long COVID should be explicitly characterized in future studies. 

We observed that compared to the effect of PW, the patients’ dyspnea was not significantly ameliorated after consuming HRW. This is inconsistent with previous studies suggesting hydrogen-rich gas (HRG) inhalation can significantly ameliorate dyspnea in COVID-19 patients [16]. This may indicate that the form of H_2_ intake is critical to its effects, especially on respiratory symptoms. Previous studies suggesting that H_2_ has benefits for dyspnea employed HRG, which can directly reduce respiratory airway flow resistance, thus alleviating dyspnea [35], while HRW is employed indirectly via the digestive system, limiting its effects on respiration. Additionally, dyspnea in long COVID may stem from cardiac implications, as suggested by Fatima and colleagues (2023). For example, they showed that people suffering from Long COVID oftentimes had myocardial inflammation and arrhythmia [36]. This cardiac involvement could exacerbate respiratory symptoms, underscoring the need for targeted H_2_ interventions. Still, no studies have directly compared the effects of HRW and HRG on functional performance in humans. Doing so may provide critical knowledge regarding the appropriate design of H_2_-based interventions for alleviating respiratory symptoms.

It was observed that greater fatigue alleviation was not significantly associated with greater improvement in cardiorespiratory endurance. This finding indicates that fatigue alleviation may not necessarily contribute to improving cardiorespiratory endurance, which is regulated by numerous factors (e.g., cardiovascular fitness and musculoskeletal systems) [37,38]. Specifically, performance in the 6MWT (e.g., total distance) may be influenced by physical activity level, quality-of-life indices, and dysfunction and the percentage of peak heart rate reached [39]. Therefore, the changes in these elements may have contributed to the observed improvement in cardiorespiratory endurance, which should be explicitly characterized in future studies, helping to reveal the underlying pathways through which HRW helps improve cardiorespiratory endurance.

Additionally, the exploratory analysis showed that sleep quality improved significantly after HRW consumption compared to PW consumption for those with difficulty sleeping. These results support the findings of previous work on the potential of H_2_ in improving sleep quality. Recent studies have shown that HRW intake has a positive effect on metabolism and aids in improving sleep quality while increasing neuronal activity in sleep-related brain regions [40,41]. During persistent inflammation induced by Long COVID, the neurons in the sleep-regulating area of the brain may be damaged, which can negatively affect circadian rhythm [42]. Therefore, HRW intake may reduce difficulty sleeping by regulating the nervous system and affecting nerve activity in sleep-regulatory regions. On the other hand, we did not observe significant improvements in mood induced by HRW compared to those provoked by PW among those with depression and/or anxiety, which may be due to the relatively small sample size of the subgroup with mood symptoms and many confounders (e.g., economic stress, family isolation, and various social contact restrictions) that may interfere with the effects of H_2_ [43,44]. Future studies with a primary focus on the effects of H_2_ on mood are thus needed to explicitly examine the effects of H_2_ on the mood symptoms of Long COVID.

This study has certain limitations. The sample size of this work is relatively small. Additionally, single blinding might have introduced bias in the observations. Future research with larger sample sizes and a more rigorous design (e.g., double blinding) is needed to examine and confirm the findings of this work. The 14-day duration of this intervention may be relatively short, and we highly recommend that studies explore the optimal number of HRW intervention sessions, as well as the corresponding dose–response relationship by using multiple assessments conducted during and after the intervention. Meanwhile, this study included vaccinated individuals; thus, the potential influence of vaccines on the effects of HRW on the symptoms of Long COVID has not been explored. Due to restrictions in place to combat the the COVID-19 pandemic, we were unable to assess the biochemical outcomes (e.g., superoxide dismutase, SOD) of the participants in person [45]. While baseline oxygen saturation levels were used as a criterion for participant inclusion, these measurements were not systematically recorded, limiting our ability to verify initial physiological conditions. Future studies are thus needed to explore the effects of HRW on these characteristics that closely pertain to COVID-19 and its related symptoms, which will ultimately advance our understanding of the underlying mechanisms through which HRW can alleviate these symptoms during infection with COVID-19. Finally, considering the anti-inflammatory and antioxidant properties of hydrogen, its effects as a supplementary treatment on post-viral fatigue, not only in Long COVID but also in other viral infections, deserve further exploration to elucidate the broader therapeutic potential of hydrogen.

## 5. Conclusions

This study demonstrated that HRW might be an effective strategy for alleviating fatigue and improving cardiorespiratory endurance, musculoskeletal function, and sleep quality. Still, it does not ameliorate dyspnea in Long-COVID patients. The knowledge provided in this study will ultimately aid in the design of effective strategies for managing symptoms (e.g., fatigue) and promoting recovery among Long-COVID patients.

## Figures and Tables

**Table 1:** Demographic and characteristics.

Characteristics	HRW (*n* = 16)	PW (*n* = 16)	*p*-Value
Age (years)	33.38 ± 11.05	38.31 ± 14.52	0.288
Height (cm)	170.06 ± 11.71	167.25 ± 8.57	0.444
Body weight (kg)	67.60 ± 15.19	64.91 ± 9.30	0.551
BMI (kg/m^2^)	23.16 ± 2.95	23.20 ± 2.82	0.934
Male/Female (%)	5/11	5/11	
Duration of COVID-19 (day)	8.44 ± 3.81	7.19 ± 3.56	0.346
Days after COVID-19 (day)	31.19 ± 6.95	32.69 ± 8.87	0.598
Difficulty in Sleeping (*n*)	9	10	
Anxiety and Depression (*n*)	9	7	

Note: Data are presented as means and standard deviations. HRW, hydrogen-rich water; PW, placebo water.

**Table 2:** Performance in all tests before and after the intervention within the HRW and PW group.

	Variable	Group (*n*)	Pre	Post	Percent Changes (%)
Fatigue	FSS (score)	HRW (16)	38.13 ± 12.71	21.69 ± 8.07 **#	−16.44 ± 11.36
		PW (16)	38.88 ± 16.35	31.44 ± 14.95	−7.44 ± 14.35
Dyspnea	mMRC (score)	HRW (16)	1.69 ± 0.70	1.19 ± 0.40 *	−0.50 ± 0.63
		PW (16)	1.63 ± 0.81	1.44 ± 0.51	−0.19 ± 0.83
Cardiorespiratory Endurance	6MWT (m)	HRW (16)	531.89 ± 96.84	601.22 ± 83.50	69.33 ± 57.10
		PW (16)	515.78 ± 98.66	534.68 ± 89.27	18.90 ± 41.54
Musculoskeletal Function	30s-CST (times)	HRW (16)	21.88 ± 6.83	27.25 ± 5.46 *#	5.38 ± 3.40
		PW (16)	22.06 ± 5.08	23.88 ± 5.54	1.81 ± 2.54
Sleep Quality	PSQI (score)	HRW (16)	6.06 ± 3.37	3.31 ± 2.89 *#	−2.75 ± 2.46
		PW (16)	4.63 ± 1.63	4.00 ± 1.83	−0.63 ± 1.41
Mood	DASS-depression (score)	HRW (16)	21.25 ± 8.10	19.00 ± 4.79	−2.25 ± 7.26
		PW (16)	18.88 ± 4.95	17.50 ± 4.76	−1.37 ± 3.91
	DASS-anxiety (score)	HRW (16)	22.75 ± 7.00	19.38 ± 4.66	−3.37 ± 7.72
		PW (16)	20.25 ± 6.06	18.25 ± 5.75	−2.00 ± 3.50
	DASS-stress (score)	HRW (16)	25.25 ± 7.86	21.63 ± 7.63	−3.62 ± 6.12
		PW (16)	20.69 ± 7.27	19.50 ± 7.17	−1.19 ± 2.17

Note: HRW, hydrogen-rich water; PW, placebo water. * Statistically significant difference between pre- and post-test (*p* < 0.05). ** *p* < 0.001. # Significant differences in the percentage changes between pre-and post-test between the HRW group and PW group (*p* < 0.05).

**Table 3:** Subgroup analysis based on difficulty sleeping or depression and anxiety.

Variable	Group (n)	Pre-Test	Post-Test	Percent Changes (%)
PSQI (score)	HRW (9)	6.89 ± 3.06	3.33 ± 3.12 *#	−3.57 ± 2.88
	PW (10)	5.50 ± 1.43	4.90 ± 1.52	−0.60 ± 1.58
DASS-depression (score)	HRW (9)	22.89 ± 10.15	19.33 ± 5.66	−3.56 ± 9.53
	PW (7)	22.29 ± 5.36	20.57 ± 5.86	−1.71 ± 5.47
DASS-anxiety (score)	HRW (9)	25.11 ± 9.75	20.00 ± 4.90	−5.11 ± 9.23
	PW (7)	25.71 ± 4.68	23.14 ± 5.64	−2.57 ± 4.58
DASS-stress (score)	HRW (9)	27.56 ± 8.53	23.33 ± 8.83	−4.22 ± 2.09 #
	PW (7)	27.14 ± 6.31	25.43 ± 7.28	−1.71 ± 2.37

Note: * Statistically significant difference between pre- and post-test (*p* < 0.05). # Significant differences in the percentage changes pre-and post-test between the HRW group and PW group (*p* < 0.05).

## Data Availability

The original contributions presented in the study are included in the article, further inquiries can be directed to the corresponding authors.
